# STATEWIDE PROGRAMS TO IMPROVE DEMENTIA DETECTION AND CARE

**DOI:** 10.1093/geroni/igae098.0421

**Published:** 2024-12-31

**Authors:** Freddi Segal-Gidan, Soo Borson

**Affiliations:** Chair; Discussant

## Abstract

States are serving as sites for innovation in dementia care and practice. Representatives from three states, Washington, Georgia and California, will discuss their respective statewide dementia programs. Each state will provide an overview of their program, its goals and accomplishments. In California, the Dementia Care Aware program focused on reaching primary care teams with a training and support program for screening, especially for Medi-Cal (Medicaid) beneficiaries. In Georgia, Georgia Memory Net, has developed a statewide network of diagnostic clinics aimed at improving the standard of dementia care in people with a diagnosis.. Finally, Washington will discuss the Cognition in Primary Care program, which developed as a quality improvement initiative for primary care that provides a framework for evaluation of cognitive concerns and tools to assist in managing dementia as it progresses..

